# Higher cartilage wear in unipolar than bipolar hemiarthroplasties of the hip at 2 years: A randomized controlled radiostereometric study in 19 fit elderly patients with femoral neck fractures

**DOI:** 10.1080/17453674.2018.1475899

**Published:** 2018-05-23

**Authors:** Wender Figved, Stian Svenøy, Stephan M Röhrl, Jon Dahl, Lars Nordsletten, Frede Frihagen

**Affiliations:** 1 Orthopaedic Department, Baerum Hospital, Vestre Viken Hospital Trust;; 2 Division of Orthopaedic Surgery, Oslo University Hospital; 3 Institute of Clinical Medicine, Faculty of Medicine, University of Oslo, Norway

## Abstract

Background and purpose — The use of unipolar hemi­arthroplasties for femoral neck fractures is increasing in some countries due to reports of higher reoperation rates in bipolar prostheses. On the other hand, it has been proposed that bipolar hemiarthroplasties have clinical advantages and less cartilage wear than unipolar hemiarthroplasties. We compared cartilage wear between bipolar and unipolar hemiarthroplasties using radiostereometric analyses (RSA), in patients aged 70 years or older.

Patients and methods — 28 ambulatory, lucid patients were randomized to treatment with a unipolar or a bipolar hemiarthroplasty for an acute femoral neck fracture. Migration of the prosthetic head into the acetabulum was measured using RSA. Secondary outcomes were Harris Hip Score (HHS), and EQ-5D scores. Patients were assessed at 3, 12. and 24 months.

Results — 19 patients were available for follow-up at 2 years: mean proximal penetration was 0.83 mm in the unipolar group and 0.24 mm in the bipolar group (p = 0.01). Mean total point movement was 1.3 mm in the unipolar group and 0.95 mm in the bipolar group (p = 0.3). Median HHS was 78 (62–96) in the unipolar group and 100 (70–100) in the bipolar group (p = 0.004). Median EQ-5D Index Score was 0.73 (0.52–1.00) in the unipolar group and 1.00 (0.74–1.00) in the bipolar group (p = 0.01). Median EQ-5D VAS was 70 (50–90) in the unipolar group and 89 (70–95) in the bipolar group (p = 0.03)

Interpretation — Patients with unipolar hemiarthroplasties had higher proximal cartilage wear and lower functional outcomes. Unipolar hemiarthroplasties should be used with caution in ambulatory, lucid patients.

For displaced femoral neck fractures, total hip arthroplasty (THA) may be the best option for healthier, active patients (Hopley et al. 2010, Burgers et al. 2012), while unipolar or bipolar hemiarthroplasty is the most common treatment in elderly patients (Miller 2013, Rogmark and Leonardsson 2016). A unipolar hemiarthroplasty (UHA) articulates between the large metal head and the acetabulum, while a bipolar hemiarthroplasty (BHA) also articulates between an inner metal head and the polyethylene of a larger head with an outer metal shell.

A systematic review of 10 randomized controlled trials (RCTs) found similar or better results for BHA compared with UHA in hip function, hip pain, and quality of life, and no differences in mortality, reoperation, dislocation, and complications. Furthermore, BHA showed less cartilage wear at 1 year, but no differences at 4 months, 2 years, and 4 years (Jia 2015). No studies have shown a clear correlation between cartilage erosion and clinical manifestations of the hip joint. 1 RCT using radiostereometric analyses (RSA) of cartilage wear in hemiarthroplasties showed increased wear in the UHA group at 2 years (Jeffcote et al. 2010). Decision-making is still difficult due to contradictory results of clinical trials, price differences in some markets, and the possibility of variances in properties between different hemiarthroplasty components.

We compared wear between a UHA and a BHA up to 2 years, using RSA and functional outcome scores, in patients 70 years and older with femoral neck fractures, with a hypothesis of equivalence between the groups.

## Patients and methods

**Figure 1.**
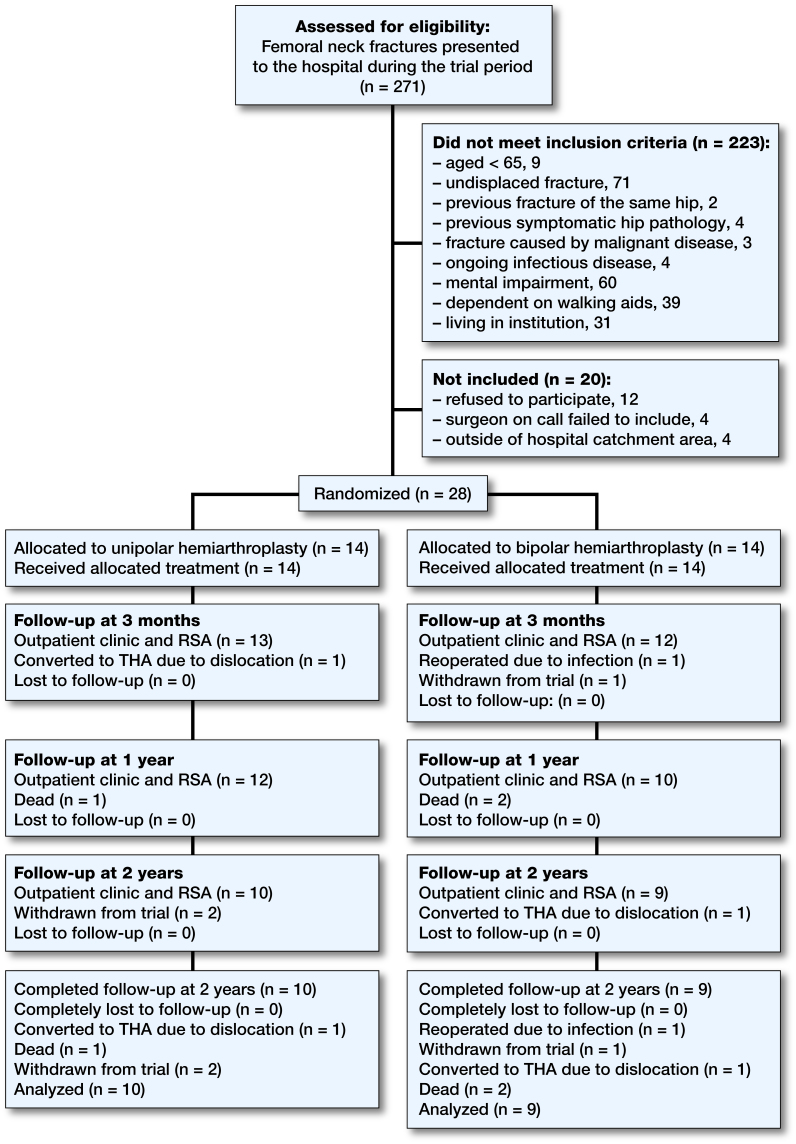
The CONSORT diagram shows the recruitment and flow of patients throughout the trial.

The trial was conducted at the orthopedic department of Baerum Hospital, Norway. Recruitment was from October 2009 to April 2011. Patients aged 70 years or older with a displaced intracapsular femoral neck fracture were eligible for inclusion. They had to be living independently and be able to walk without aids. Patients with cognitive impairment, previous symptomatic hip pathology such as osteoarthritis, a fracture caused by malignant disease, or ongoing infectious disease were excluded. Randomization was performed using a computer random number generator. Allocation was done by the surgeon on call using sealed envelopes. 28 patients were randomized to treatment with a cementless UHA or BHA for an acute femoral neck fracture (Table 1). Patients were followed at 3 months, 1 year, and 2 years. 19 patients were available for follow-up at 2 years (Figure 1).

**Table 1.** Patient characteristics at the time of enrollment

Characteristics	Unipolar (n = 14)	Bipolar (n = 14)
		
Median age (range)	81 (70–90)	80 (70–89)
Female sex	11	11
Mean preoperative Harris Hip Score (SD)	94 (6)	96 (4)
Mean preoperative EQ-5D Index (SD)	0.90 (0.12)	0.91 (0.11)
Mean preoperative EQ-5D VAS (SD)	72 (17)	79 (16)
Median outer head size (range)	49 (45–53)	48 (46–54)
		

## Intervention

**Figure 2.**
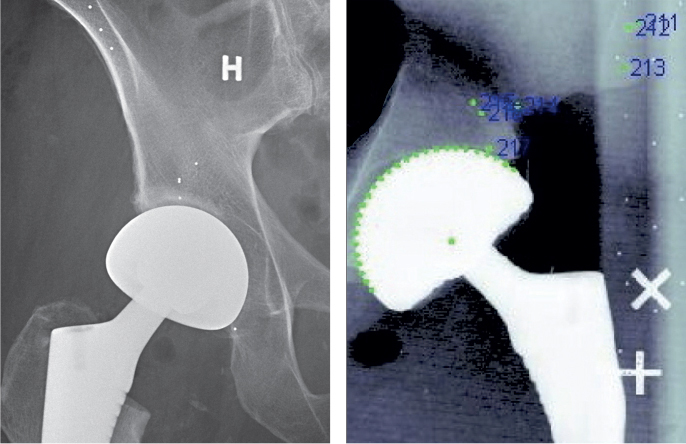
The image on the left is a conventional radiograph showing a bipolar hemiarthroplasty of the right hip. Tantalum markers have been implanted around the acetabulum, in the anterior superior iliac spine, and in the superior pubic ramus. To the right is a radiostereometric image with tantalum markers in the pelvis (numbered) and tantalum markers in the calibration cage. The center of the outer prosthetic head has been computer-calculated using edge detection.

Patients were operated with a hemiarthroplasty using an uncemented press-fit hydroxyapatite-coated femoral stem (Corail, DePuy Orthopaedics Inc, Warzaw, IN, USA). The BHA group received a 28 mm cobalt chromium head and a bipolar head (Self-Centering™ Bipolar, DePuy Orthopaedics Inc, Warzaw, IN, USA). The UHA group received a modular unipolar head (Modular Cathcart Unipolar, DePuy Orthopaedics Inc, Warzaw, IN, USA). Both head options were available in 1 mm size increments. The diameter of the femoral head was measured using full circular measurement templates during surgery (Jeffery and Ong 2000), and the corresponding prosthetic head size was chosen (Table 1). Arthroplasty was performed through a posterior approach with the patient in the lateral decubitus position, using spinal anesthesia. 5 or 6 1 mm tantalum (Ta) spherical markers were inserted in the pelvis around the acetabulum, and 3 in the anterior superior iliac spine, using an UmRSA Injector (RSA BioMedical, Umea, Sweden) (Figure 2). 6 experienced surgeons conducted the procedures. All patients were given preoperative intravenous cefalotin 2 g and a further 3 doses in the first 12 hours after the operation. All patients received 5000 IU low molecular weight heparin subcutaneously daily for at least 10 days. Early mobilization was encouraged, with weight bearing as tolerated.

## Outcomes and data collection

The primary outcome was migration of the prosthetic head into the acetabulum. Migration was measured with UmRSA software (RSA BioMedical, Umea, Sweden) using an RSA cage 43 containing Ta markers for creation of 3D coordinates and built-in film cassette holders placed behind the patient. Radiostereometric examinations were conducted using 2 fixed X-ray tubes angled approximately 40 degrees in relation to each other. The center of the outer head was determined by semi-automatic edge detection of the metal shell in the BHA group, and the surface of the unipolar head in the UHA group; 1 experienced analyzer localized the edge with 4 points, and the software automatically detected the remaining edge points according to the pixel difference (Borlin et al. 2006, Figved et al. 2012) (Figure 2). The motion of the center of the outer head was calculated relative to the rigid body segment created by the Ta markers in the pelvis, in all 3 planes. Although published after study completion, the trial complies with the ISO standard for RSA (ISO copyright office 2013). However, for comparison with previous trials, and clinical relevance, proximal penetration (Y-axis) and the total point movement (TPM) of the femoral head were reported as a surrogate for wear of the acetabular cartilage.

The RSA index radiographs were taken within 1 week postoperatively. To determine the precision of the RSA measurements, all examinations were conducted in the supine position and repeated within 1 hour, with repositioning of the patient between the scans. The precision was then calculated from the mean difference between the double examinations at all time intervals. For analyses of cartilage wear, double examinations of all patients at all time intervals were compared, and the mean result of the analyses was recorded. Hip function was rated with Harris Hip Score (HHS), ranging from 0 to 100 points covering a maximum of 44 points for absence of pain, 47 points for function, and 9 points for range of motion and absence of deformity. Health-related quality of life was rated by the patient-reported EQ-5D using VAS and index scores. After inclusion but prior to surgery, all patients completed an HHS and an EQ-5D, instructed to recall and assess their pre-fracture status (Table 1).

## Statistics

Sample size calculation was conducted using the equivalence criterion and the extension of the CONSORT statement on non-inferiority and equivalence trials, and was based on an assumed precision of 0.2 mm of our RSA measurements. With no previously published values for a clinically relevant difference in cartilage wear, we chose an arbitrarily selected difference of 0.5 mm. A sample size of 6 patients in each group was calculated to be sufficient, with a 2-sided 95% confidence interval (CI) and 95% power, to establish equivalence. A margin of equivalence of 0.5 mm and a range of –0.5 to 0.5 was predefined as an acceptable range for the CI of the difference in wear. To compensate for loss to follow-up, complications, and mortality, we decided to include 28 patients. To avoid analyzing RSA measurements of patients that were converted to a total hip arthroplasty or reoperated for infection, a per-protocol design was used. For RSA analyses, HHS and EQ-5D scores, we used the nonparametric independent-samples Mann–Whitney U test. SPSS version 24 for Macintosh (IBM Corp, Armonk, NY, USA) was used for statistical analyses.

## Ethics, registration, funding, and potential conflicts of interests

The protocol was approved by the regional ethics committee (S-08619b) and registered at Clinicaltrials.gov (NCT00746876). Patients provided written informed consent prior to surgery. The study was conducted in compliance with the Helsinki Declaration, and the CONSORT Statement. The study was founded by the 2 participating hospitals. The first author received a research grant for this study of NOK 50,000 from Smith & Nephew, Norway. There are no other conflicts of interest to be reported by any of the authors.

## Results

The precision of the measurements expressed by the mean difference between 91 double examinations was 0.029 mm for the X-axis (99% CI –0.007 to 0.065), 0.028 mm for the Y-axis (99% CI –0.005 to 0.060), and 0.009 mm for the Z-axis (99% CI 0.04 to 0.06).

The distribution of the markers in the pelvis was assessed using the condition number which was below 150 in all but 3 examinations in 3 different patients, which were then excluded from analyses (mean 119; median 66 (25–1387)). The stability of the markers was assessed using the mean error of rigid body fitting, which was below 0.35 in all cases (mean 17; median 0.17 (0.006–0.345)) (ISO copyright office 2013).

**Figure 3.**
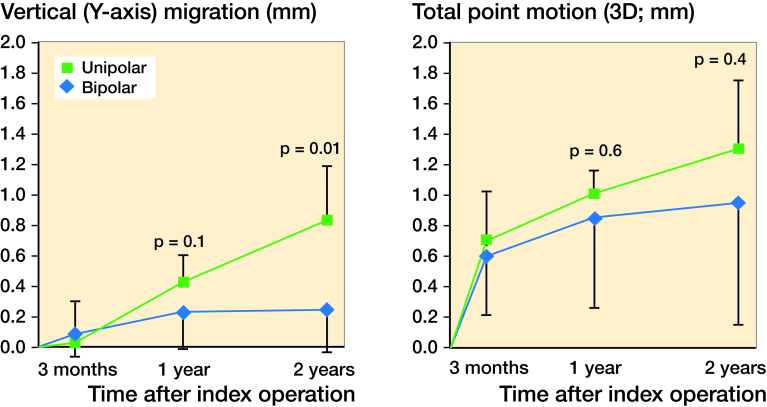
Graph showing mean migration in the vertical plane (Y-axis) and total point motion (3D migration) of patients at 3 months, 1 year, and 2 years.

**Figure 4.**
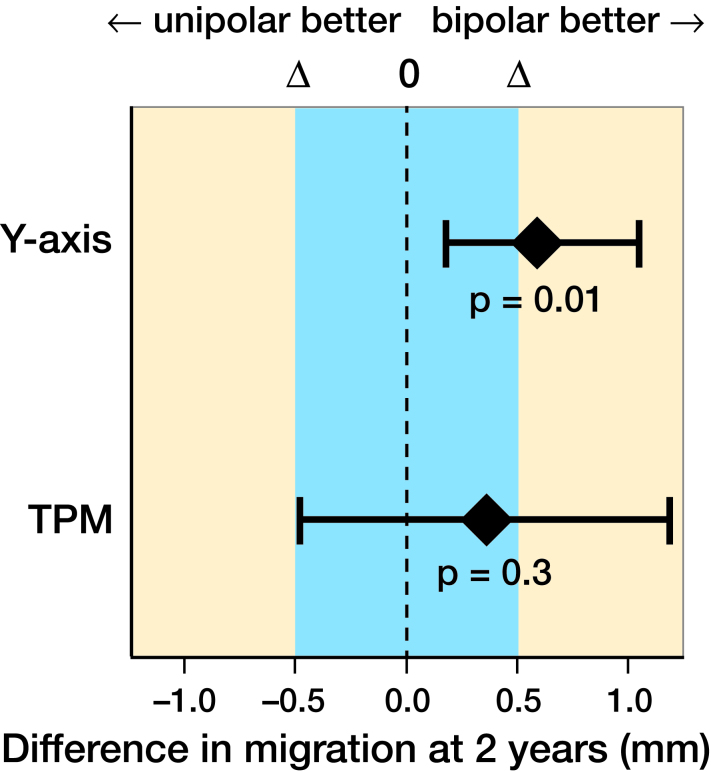
The graph shows the mean difference of proximal penetration (Y-axis) and TPM between the two groups at 2 years, in millimeters. Error bars indicate 95% confidence intervals (CIs) for the mean difference. Blue area indicates zone of equivalence, defined as ±0.5 mm (Delta). The CI for the Y-axis lies above zero but exceeds the zone of equivalence, indicating a superior result. For TPM, the 95% CI includes zero and exceeds the zone of equivalence, indicating a nonsignificant result.

Mean proximal penetration (Y-axis) at 3 months was 0.023 mm in the UHA group and 0.083 mm in the BHA group (CI –0.4 to 0.2), at 1 year 0.43 mm in the UHA group and 0.23 mm in the BHA group (CI –0.07 to 0.5), and at 2 years 0.83 mm in the UHA group and 0.24 mm in the BHA group (CI 0.1 to 1.0) (Figure 3). The CI interval for the mean difference at 2 years was above zero but exceeded the equivalence margin of 0.5 mm, indicating a superior, and not equivalent, result (Figure 4).

Mean TPM at 3 months was 0.71 mm in the UHA group and 0.60 mm in the BHA group (CI –0.4 to 0.6), at 1 year 1.0 mm in the UHA group and 0.86 mm in the BHA group (CI –0.5 to 0.7), and at 2 years 1.3 mm in the UHA group and 0.95 mm in the BHA group (CI –0.4 to 1.1) (Figure 3). The CI interval for the mean difference at 2 years included zero and exceeded the equivalence margin of 0.5 mm, indicating a statistically nonsignificant result (Figure 4).

**Figure 5.**
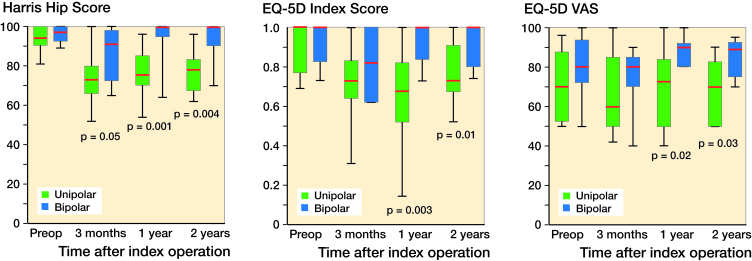
Box plots of Harris Hip Score, EQ-5D Index Score, and EQ-5D Visual Analogue Scale (VAS) at all time intervals. Boxes represent the middle 50% of the data, with the central band representing the median. The ends of the whiskers represent the minimum and maximum of all of the data. Statistical analyses conducted using the non-parametric Mann–Whitney U test.

Median HHS, EQ-5D Index Score and EQ-5D VAS was higher in the BHA group at all time intervals, and statistically significantly higher at 2 years (Figure 5).

## Discussion

In this trial, patients treated with UHA had higher proximal cartilage wear. The wear rate corresponds well with the only previously published similar RSA trial (Jeffcote et al. 2010). TPM, mediolateral (X-axis), and anteroposterior (Z-axis) migration were similar between the groups. Thus, we only detected a difference in cartilage erosion in the proximal direction (Y-axis), corresponding to wear of cartilage in the dome of the acetabulum. The Y-axis might be the best way of measuring early cartilage erosion, being the loadbearing direction. Jeffcote et al. (2010), also found differences in TPM at 1 and 2 years’ follow-up, favoring BHA. 1 RCT found more radiological cartilage wear in the UHA group during the first 12 months. The difference diminished over time, and was no longer statistically significant at 2 years and 4 years. The study also suggests the mechanism of the BHA ceases to function after some time, and behaves like a UHA (Inngul et al. 2013). This was also proposed in earlier studies (Chen et al. 1989, Eiskjaer et al. 1989).

A recent meta-analysis of RCTs comparing unipolar versus bipolar hemiarthroplasty for displaced femoral neck fractures did not find an advantage of bipolar prostheses (Jia 2015). However, the review lacks information on whether the same femoral stem was used in both groups of the included trials. Two recent RCTs including hemiarthroplasties did not list revision due to cartilage wear as a problem, during 5 to 7 years’ follow-up (Støen et al. 2014, Langslet et al. 2014). Although many patients in these studies did not have a late radiograph taken, the follow-up was good, and patients in pain were addressed.

The difference in HHS, EQ-5D Index, and VAS scores in this trial should be interpreted with caution. The sample size calculation conducted for this study lacks power for these secondary outcomes, and they should therefore be considered subsidiary, with a high risk of a Type 1 error—a false-positive result. We found a surprisingly large difference in favor of the BHA group in all secondary outcomes at 1 and 2 years. In our trial, prosthetic head migration was the primary outcome measure, and the sample size is too low to show a trustworthy difference in any of the functional outcome scales used. Our trial, however, recruited a fit subgroup of patients with femoral neck fractures, so good clinical results would be expected (Hebert-Davies et al. 2012, Mundi et al. 2014).

Decision-making is still difficult due to contradictory results of clinical trials, and the possibility of variances in properties between different hemiarthroplasty components: The first step towards a bipolar hemiarthroplasty was introduced by Christiansen in the late 1960s (Christiansen 1969). This prosthesis had a built-in trunnion bearing that allowed some movement between the stem and the head of the prosthesis. The results were promising (Soreide et al. 1975, Meyer 1981), but acetabular protrusion remained a problem (Søreide et al. 1980). The first true bipolar model with a ball and socket joint between the femoral stem and the prosthetic head was the Bateman (1974) hemiarthroplasty. The bipolar design was then used in similar models such as the Giliberty, Monk, and Hastings. Many series with short- and long-term follow-up showed less pain and decreased protrusion of the acetabulum than in previous reports on UHA (Devas and Hinves 1983, LaBelle et al. 1990, Wetherell and Hinves 1990, Haidukewych et al. 2002, Isotalo et al. 2002). However, no randomized controlled trials comparing UHA with the newer BHA models were conducted until much later.

Early radiological studies of interprosthetic motion in bipolar hemiarthroplasties showed little or no movement between the stem and the head over time when analyzing passive motion of the hip without weight-bearing (Bochner et al. 1988, Hodgkinson et al. 1988, Chen et al. 1989). Later studies analyzing the interprosthetic movement during weight-bearing have, however, shown a preserved movement of the inner joint during the stance phase of gait (Wada et al. 1997, Gaine et al. 2000). One recent RSA study has shown steady-state wear over time (Tsukanaka et al. 2017).

Cartilage wear may also be measured by the rate of revision surgery. In a Swedish register study, Leonardsson et al. (2012) found a lower risk for reoperations caused by erosion in the bipolar HA, though the total revision rate was very low (0.17%). Counting all reasons for revision surgery, they found a higher risk of early reoperation following bipolar hemiarthroplasty compared with unipolar. The Australian National Joint Replacement Registry nonetheless found that bipolar prostheses had a decreased risk of revision than unipolar, at least in younger patients (Rogmark and Leonardsson 2016). In the study from Inngul et al. (2013), there was no difference in revision rates between unipolar HA and bipolar HA. There was also no correlation between cartilage wear and clinical scores (EQ-5D index score and HHS). Baker et al. (2006) found frequently radiological erosion in UHAs in lucid patients. Still, only a few were surgically revised. In a Cochrane review including 7 trials (857 participants, 863 fractures), no differences were found between UHA and BHA. The review analyzed clinical scores and complications. However, several of the studies included few patients (Parker et al. 2010). Variations in inclusion criteria may influence outcomes: It would be reasonable to stipulate that using UHA in community walking individuals (Baker et al. 2006) would certainly increase the rate of wear, compared with studies on those with very limited walking ability.

Several authors comparing UHA and BHA have discussed the issue of price differences between the 2, with the BHA usually being the more expensive implant. In our trial, however, the bipolar head plus the inner head used in the BHA group had a lower price than the unipolar head used in the UHA group. Also, when this study started, the use of THA in hip fracture patients was not common. Today, our study population of individuals living independently and able to walk without aids is not the group recommended to have unipolar hemiarthroplasties, but rather THA (Hopley et al. 2010, Burgers et al. 2012).

In summary, we found that patients treated with BHA had lower proximal cartilage wear than patients with UHA. The BHA group showed superior clinical outcomes, but an uncertain observation because of few patients. Unipolar hemiarthroplasties should be used with caution in self-ambulatory, lucid patients.

Idea conception: WF, LN. Inclusion, surgery, and follow-up: WF, SS. Implementation of RSA technique: JD. RSA analyses: SR. Data analyses. WF, SS, JD, LN, FF. Manuscript preparation: all authors.
Thanks are offered to Gunhild Olseng and Elisabeth Gunby for radiographic and RSA services in accordance with the highest standards, and to Jan Erik Madsen, Finnur Snorrason, and Asbjørn Hjall for important parts in conceiving the idea and facilitating this trial. 
*Acta* thanks Lennard Koster and Cecilia Rogmark for help with peer review of this study.
